# Effect of Freeze-Thaw Cycles on Juice Properties, Volatile Compounds and Hot-Air Drying Kinetics of Blueberry

**DOI:** 10.3390/foods10102362

**Published:** 2021-10-04

**Authors:** Lin Zhu, Xianrui Liang, Yushuang Lu, Shiyi Tian, Jie Chen, Fubin Lin, Sheng Fang

**Affiliations:** 1 School of Food Science and Biotechnology, Zhejiang Gongshang University, Xuezheng Street No. 18, Hangzhou 310018, China; wishzzl@163.com (L.Z.); lyszjgsl@163.com (Y.L.); tianshiyi@zjgsu.edu.cn (S.T.); chenjie@zjgsu.edu.cn (J.C.); linfubinwin@126.com (F.L.); 2 Collaborative Innovation Center of Yangtze River Delta Region Green Pharmaceuticals, College of Pharmaceutical Sciences, Zhejiang University of Technology, Hangzhou 310014, China; liangxrvicky@zjut.edu.cn

**Keywords:** freeze-thaw cycles, anthocyanins, gas chromatography-mass spectrometry, aroma profiles, hot-air drying, blueberry

## Abstract

This paper studied the effects of freeze-thaw (FT) cycles on the juice properties and aroma profiles, and the hot-air drying kinetics of frozen blueberry. After FT treatment, the juice yield increased while pH and total soluble solids of the juice keep unchanged. The total anthocyanins contents and DPPH antioxidant activities of the juice decreased by FT treatments. The electronic nose shows that FT treatments significantly change the aroma profiles of the juice. The four main volatile substances in the fresh juice are (E)-2-hexenal, α-terpineol, hexanal and linalyl formate, which account for 48.5 ± 0.1%, 17.6 ± 0.2%, 14.0 ± 1.5% and 7.8 ± 2.7% of relative proportions based on total ion chromatogram (TIC) peak areas. In the FT-treated samples, the amount of (E)-2-hexenal and hexanal decreased significantly while α-terpineol and linalyl formate remained almost unchanged. Repeated FT cycles increased the ethanol content and destroyed the original green leafy flavor. Finally, the drying kinetics of FT-treated blueberries was tested. One FT treatment can shorten the drying time by about 30% to achieve the same water content. The *D_eff_* values of the FT-treated sample are similar, which are about twice as large as the value of the fresh sample. The results will be beneficial for the processing of frozen blueberry into juice or dried fruits.

## 1. Introduction

Blueberry is widely grown all over the world and its production has largely increased in recent years [1]. Due to seasonality and short shelf life, approximately 50% of blueberries are processed into food products such as juice and dry fruits [2]. Most of the blueberries used for juice processing and drying are frozen fruits. During storage and cold chain transportation, frozen blueberries might be subjected to several freeze-thaw (FT) cycles [3]. Insights into the changes of frozen blueberries during repeated FT cycles are essential for the processing of blueberry products [4].

FT treatment will affect the qualities and flavors of final food products [5,6]. The ice crystal formed in FT treatments facilitates the rupture of fruit cell walls and also changes the texture and flavor during the thawing process. For blueberries, the study has shown that FT treatment facilitated moisture transfer during drying and produced softer, less chewable and less gummy berries [7]. The juice yield of fruits and extraction efficiency of bioactive compounds were also improved by the FT treatments of blueberries. However, Nowak et al. [8] found that FT treatments induced considerable changes in the color of blueberries. Yan et al. [9] found that aroma deterioration during subsequent shelf life after cold storage was more fatal to the blueberry flavor. So, the FT treatment exerts both desirable and undesirable effects on the physicochemical properties of blueberry [10]. To the best of our knowledge, there are no studies on the effects of multiple FT cycles on the physicochemical properties of frozen blueberry, especially focus on, aromatic compounds and drying kinetics.

In this study, the effects of FT cycles on the physicochemical properties and volatile compounds of blueberry juice, and hot-air drying kinetics of frozen blueberry fruits were studied. The juice yields, pH, total soluble solids, total anthocyanin contents and antioxidant properties of the juice were firstly characterized. Then, the electronic nose and gas chromatography-mass spectrometry (GC-MS) was used to study the influences on aroma profiles. Finally, the kinetic characteristics of hot-air drying of blueberry at 60 °C were studied, and the drying model and parameters were obtained. The results will be beneficial for the further processing of blueberry frozen fruit into juice and dried fruits.

## 2. Materials and Methods

### 2.1. Materials

Blueberry which belongs to a southern-highbush variety was harvested at the mature stage (fully blue and firm) from a local orchard in Zhuji, Zhejiang Province, China. Fruit without disease and wounds were harvested. Chromatography grade acetonitrile, methanol, ethanol, formic acid (purity ≥ 98%) and acetic acid (purity ≥ 99%) was purchased from Aladdin (Shanghai, China). All other reagents were of analytical grade.

### 2.2. Freeze-Thaw Treatments

Blueberries were washed with water and put in a sealed bag. For each bag, a total of 60 blueberries were carefully selected without physical damage and used. Three bags were set as one group for each FT treatment. The blueberry in the bag was frozen at −25 °C [11] for 6 h in a low-temperature refrigerator (MDF-330, SANYO, Japan) and then thawed at 4 °C for 3 h. FT-1, FT-2 and FT-3 represent the sample with FT treatment once, twice and third times. After FT treatments, the blueberry was juiced with a juicer. The juice was centrifuged at 10,000 rpm and 20 °C for 15 min. The supernatant was taken and analyzed.

### 2.3. The pH and Total Soluble Solids (TSS)

The pH values of blueberry juices were obtained using a pH meter (PHS-3G, Shanghai INESA, China) at 25 °C. TSS was determined by measurement of refractive index at 25 ± 1 °C using a digital refractometer (WAY-2S, Shanghai Shengke, Shanghai, China).

### 2.4. Total Anthocyanins Content by the pH Differential Method

The anthocyanin content in the juice was determined using the pH differential method [12]. The content was expressed based on cyanidin-3-O-glucoside (*C3G*). The absorbance (*Ab*) was measured using a UV spectrophotometer (UV-2600, Shimadzu, Japan) at 510 and 700 nm. The total anthocyanins (TA) are calculated as:

(1) $$\mathrm{TA}\;(\mathrm{mg}/L)=\frac{\Delta Ab\times M\times Df}{L\times \varepsilon }$$

where Δ*Ab* is calculated as (*Ab*_510_-*Ab*_700_)_pH=1.0_-(*Ab*_510_-*Ab*_700_)_pH=4.5_; *M* is the molar mass of *C3G*, 449.2 g/mol; *Df* is the dilution factor; *L* is the path length in cm; *ε* is the molar extinction coefficient of *C3G*, 26900 L/mol/cm.

### 2.5. DPPH Free Radical Scavenging Test

The free radical scavenging ability was measured according to the method of Kahkonen [13] with some modifications. 100 mL ethanol solution contains 0.1 mM DPPH was prepared and stored in a refrigerator at 4 °C. Incubate 2 mL sample solution and 2 mL ethanol DPPH solution (0.1 mM) at room temperature (25 °C) for 0.5 h. Finally, the absorbance of the mixture was measured at 517 nm using a UV spectrophotometer (UV-2600, Shimadzu, Japan). As a control, distilled water was used instead of DPPH.

### 2.6. Electronic Nose Test

The sensory profiles of the blueberry juice were tested by an electronic nose according to a previous study [14]. The juice sample was put into a 500 mL beaker, wrapped and sealed with tin foil, and the headspace was allowed to balance for 30 min at room temperature. The equipment was exhausted for 120 s at first. Then, the headspace gas of the beaker was pumped through the sensor array at a rate of 0.6 L/min. The measurements lasted for 120 s to ensure a stable response [15].

### 2.7. Volatile Compounds Profile Analyzed by GC-MS

The volatile compounds were analyzed according to a previous study [16]. An Agilent 7890B-5977B gas chromatograph-mass spectrometer equipped with an Agilent 7697A Headspace auto-sampler (Santa Clara, CA, USA) was used. A capillary column (DB-624 UI, Agilent Technologies, Santa Clara, CA, USA) of 30 m × 0.25 mm with 1.4 μm film was selected. The blueberry juice (3 mL) and saturated NaCl solution (1 mL) were mixed into a 20 mL headspace bottle for the GC-MS test. The headspace equilibrium temperature and time are 80 °C and 40 min, respectively. The quantitative loop and transmission line temperature are set at 90 °C and 100 °C, respectively. Helium (>99.9%, 1 mL/min) was used as carrier gas. The sample was injected into the GC column in a split ratio (1:5) mode. The initial column temperature is set at 35 °C and kept for 2 min. Then increase to 190 °C at a rate of 4 °C/min. Then increase to 240 °C at a rate of 8 °C/min and hold for 5 min, and finally cool to 35 °C. The MS spectrum was obtained at 70 eV in electron ionization (EI) mode with the scanning range 35–600 m/z. The MS ion source and detector temperatures are 230 °C and 260 °C, and the solvent delay time is set to 2 min. The compound is identified by comparing the mass spectrum with the reference mass spectrum in the NIST 14. The relative content of each compound was calculated based on the peak area of total ion chromatograms (TIC). Each sample was measured in duplicate, and the values were presented as mean ± standard deviation.

### 2.8. Hot-Air Drying Conditions and Modeling

#### 2.8.1. Hot-Air Drying Conditions

The drying experiments were conducted by a hot-air convective dryer that is the same as a previous study [17]. The dryer was set at 60 °C with an air velocity of 1.2 m/s and was equilibrated for at least 1 h. A single layer of blueberries (about 14 g) was placed on stainless steel wire meshes in the dryer that weighted in real-time with an electronic balance. The sample weight was recorded every 5 min for 10 h. After drying, the sample continues to be dried to a constant weight at 110 °C [18] in an oven, and the final mass is taken as the absolute dry mass of the sample (*W_d_*). The moisture ratio (*MR*) at time t in the drying process is calculated as follows:

(2) $$MR=\frac{W_{t}-W_{d}}{W_{0}-W_{d}}$$

where *W_t_,* and *W*_0_ represent the mass of sample at time *t*, and the initial mass of the sample, respectively.

#### 2.8.2. Mathematical Models

Nine empirical equations (the Newton, Page, modified Page, Henderson and Pabis, Logarithmic, two-term, two-term exponential, Wang and Singh, and diffusion approaches) [17] used to fit the experimental data are listed in Table S1. The model parameters were obtained by fitting the experimental drying values with the nonlinear least square method. The determination coefficient (*R*^2^) and root mean square error (*RMSE*) were calculated to test the fitting performance of each model.

The effective moisture diffusivity coefficient (*D_e_*) is an important transport property. Based on the general solution of Fick’s second law of diffusion and assumptions for spherical particles, the following equation can be obtained:

(3) $$\ln MR=\ln\left(\frac{6}{\pi^{2}}\right)-\pi^{2}\left(\frac{D_{e}}{R_{p}^{2}}\right)t$$

where *R_p_* is the average radius of spherical particle (m). As with most calculations of logarithmic kinetic models, a linear plot of ln*MR* versus time *t* was used to obtain the value of *D_e_* [19].

### 2.9. Statistical Analysis

All experiments were conducted at least in triplicate, and data were analyzed by Origin 2018 software (Origin Lab Corporation, USA). The obtained results were statistically evaluated by ANOVA single factor analyze (*p* < 0.05) with Duncan’s multiple comparisons using SPSS 19.0 software (SPSS Inc, Chicago, IL, USA).

## 3. Results and Discussion

### 3.1. Effect of FT Cycles on the Physicochemical Properties of Blueberry Juice

Table 1 shows the effects of FT cycles on the juice yield, pH and, TSS of the blueberry juice. The dry and wet water content of fresh blueberries studied are 6.3 ± 0.4 kg H_2_O/kg db (dry basis) and 86.3 ± 0.7%, respectively. The juice yield of fresh blueberries is approximately 50%, while the sample after FT treatment is approximately 60%. The increase in FT cycles did increase the juice yield as compared to the fresh one (*p* < 0.05). The FT treatment causes the cells to rupture and therefore makes the release of cellular juice easier [20,21]. The pH and TSS of the fresh juice are 3.64 ± 0.02 and 12.33 ± 0.33, respectively. The results are within the value range of fresh blueberry juice [22]. However, there are no significant differences in the pH and TSS of blueberry juice with FT treatments. Similar results were also found in the effects of FT treatment on carrot juice [23].

**Figure 1:**
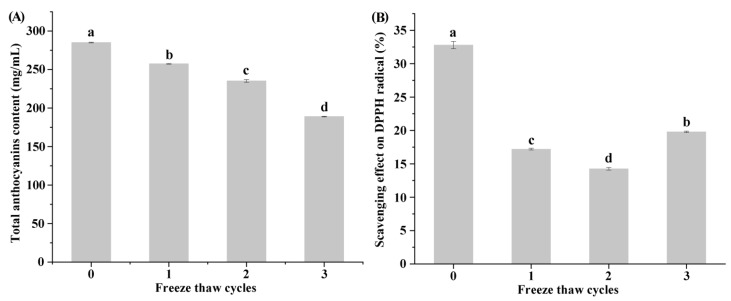
The effects of freeze thaw cycles on (**A**) the total anthocyanins content (mg/mL), and (**B**) the DPPH radical scavenging (%). Values are presented as mean ± standard deviation (*n* = 3). Different letters indicate significant differences (*p* < 0.05).

Figure 1A shows the effects of FT cycles on the total anthocyanins content of the blueberry juice. The total anthocyanins content in the initial blueberry juice is 285.0 ± 0.5 mg/L and is lower than the literature values that by ethanol extraction methods [24,25]. It might be attributed to the fact that anthocyanins were not fully extracted by the juicing process in this study and remained in the pomace [26]. The total anthocyanins decreased significantly (*p* < 0.05) after repeated FT treatment. After one, two and three FT cycles, the total anthocyanins in the juice decreased by 9.7%, 17.5% and 33.7%, respectively. Anthocyanins are unstable and are easily degraded by free radicals and enzymes [26]. Cell structure damage caused by the FT treatments leads to the leak of enzymes such as polyphenol oxidase easier, thereby improved the enzyme-substrate interaction [27]. Similar results were also found in the effects of FT treatment on cherry anthocyanins [27].

Figure 1B shows the effects of FT cycles on the antioxidant capacity of the juice. Fresh blueberry juice has the strongest antioxidant capacity. As the number of FT cycles increases, the antioxidant capacity of the samples first decreased and then slightly increased. However, after three FT cycles, the antioxidant capacity is only about 50% of that for the fresh sample. It is known that the anthocyanins content is positively related to DPPH antioxidant capacity [28]. The degradation of anthocyanins in the blueberry may result in the reduction of antioxidant activity. However, the DPPH antioxidant activity is not only determined by anthocyanins, but also by many other compounds including the products of degradation. The formed compounds might have higher activities than the original ones. The study also found that the antioxidant effect of juices containing anthocyanins and other phenolic compounds is increased after biotransformation [29].

### 3.2. Effect of FT Cycles on the Aroma Profiles of Blueberry Juice

**Figure 2:**
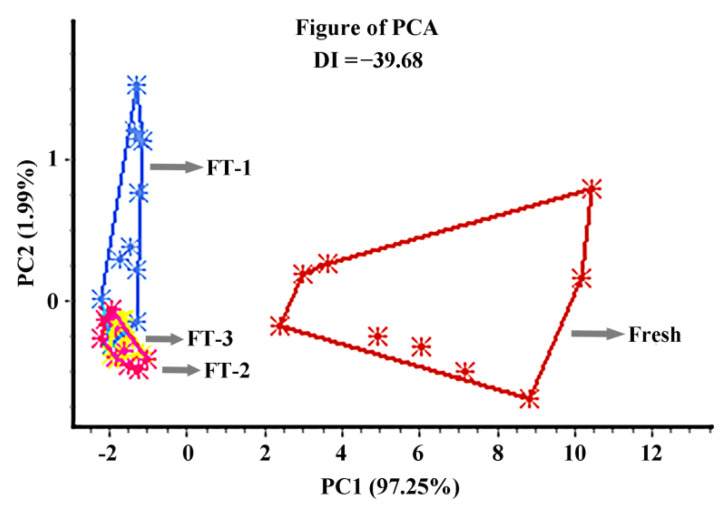
The principal component analysis (PCA) results of freeze-thaw (FT) treated samples. PC1 and PC2 represent the first and second principal component, respectively; FT-1, FT-2 and FT-3 represent the sample with FT treatment once, twice and third times.

The aroma profiles of the juice samples were first evaluated by electronic nose. PCA visualization for the electronic nose was shown in Figure 2. The first and second principal components accounted for 97.25% and 1.99%, respectively, which explained 99.24% of the variance. Fresh samples and freeze-thaw processed samples can be distinguished well. The abscissas of fresh samples and FT processed samples are far apart, which shows the good discrimination of different samples. Samples by FT once and 2 times can also be distinguished to a certain extent, but the difference is small. Nevertheless, the overlap observed at samples of 2 and 3 FT cycles indicated that these samples might have similar aroma profiles. Overall, the electronic nose shows differences for different FT treated samples [30].

The typical GC-MS total ion chromatograms (TIC) of fresh and frozen blueberry juice are shown in Figure S1 (supporting information). Most volatile compounds can be separated under the chromatographic conditions in this study. Many gas chromatographic conditions including the headspace parameters were compared. It is worth pointing out, without adding saturated NaCl, the peaks in TIC are relatively weak. When 1 mL of saturated NaCl solution is added to 3 mL of blueberry juice, the number of peaks and signal intensity are greatly enhanced. The chemical structure of most of the peaks in TIC can be obtained by matching the mass spectrum with the NIST database, and the reported flavor substances of blueberry in literatures [31,32].

**Figure 3:**
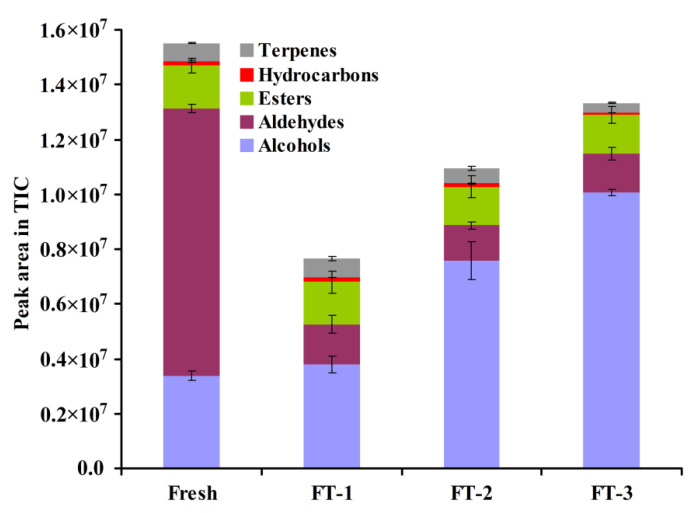
Abundance of peak area in TIC assigned to different class of volatiles for different samples. FT-1, FT-2 and FT-3 represent the sample with FT treatment once, twice and third times.

A total of 28 main volatiles are identified and grouped into five groups, including 10 alcohols, 4 aldehydes, 3 esters, 2 hydrocarbons and 9 monoterpenes as shown in Table 2. Figure 3 shows the average profiles of peak area abundance on TIC and the distribution of each volatile class. Three mainly volatile substance classes in the fresh juice are aldehydes, alcohols and esters. The relative proportions of aldehydes, alcohols and esters calculated from TIC peak areas are approximately 62.7 ± 0.4%, 21.7 ± 1.0% and 10.1 ± 1.8%, respectively. The peak area values of volatile compounds in the fresh juice and FT treated samples are shown in Figure S2. The four main volatile substances in the fresh juice are (E)-2-hexenal, α-terpineol, hexanal and linalyl formate, which account for 48.5 ± 0.1%, 17.6 ± 0.2%, 14.0 ± 1.5% and 7.8 ± 2.7%, respectively. The result is similar to the literature that the aroma profiles of blueberry are mainly aldehydes including (E)-2-hexenal and hexanal, and additional relatively high amounts of terpene compounds [31,32]. It is known that the (E)-2-hexenal has a relatively low odor threshold of 17 ppb with a green leafy aroma descriptor [33]. Esters of linalool such as linalyl formate are responsible for the fruity aroma descriptor [34]. The results are consistent with the sensory evaluation of the fresh blueberry sample.

**Figure 4:**
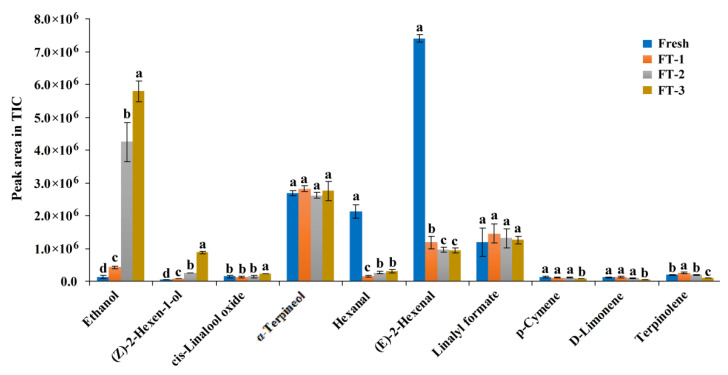
The peak area in total ion chromatogram (TIC) assigned to 10 main compounds for freeze-thaw (FT) treated samples. FT-1, FT-2 and FT-3 represent the sample with FT treatment once, twice and third times. Values are presented as mean ± standard deviation (*n* = 2). Different letters indicate significant differences (*p* < 0.05).

Figure 3 shows that the total amount of volatile substances in the juice is reduced overall after FT treatments. This is also consistent with the result of the weakening of the flavor after FT treatments in the sensory evaluation. Figure 4 shows the changes in the peak area abundance of 10 main aroma compounds in different samples. In the FT treated samples, even if only once (FT-1), the amount of C6 aldehydes including (E)-2-hexenal (*p* < 0.001) and hexanal (*p* < 0.01) decreased significantly as compared to that in the fresh juice. The amount of hexanal and (E)-2-hexenal in juice decreased approximately 93.2 ± 0.4% and 84.0 ± 2.8%, respectively, by only one FT treatment. Even though not very obvious, an increase in (Z)-2-hexen-1-ol was observed at the same time. Interestingly, the amount of α-terpineol and linalyl formate remained unchanged after FT cycles and become the main aroma compounds other than C6 aldehydes. Therefore, we speculate that the weakening and changes in the flavor of FT treated blueberries are mainly due to the degradation of C6 aldehydes. It is supposed that these C6 aldehydes might be converted into C6 alcohols during the thawing process by enzymatic reduction. During the process, the cell structure changes which might facilitate the release of enzymes such as alcohol dehydrogenase and lipoxygenase [35]. In the fermentation process of some grape wines, the study found that C6 aldehydes (hexanal and trans-2-hexenal) decreased significantly during the process [36].

In the samples after multiple FT cycles, the ethanol content increased significantly. Moreover, the increase in ethanol content has little correlation with the decrease in other flavor substances. On the other hand, the samples that were FT treated 2 and 3 times showed little change in other flavor substances except for ethanol and (Z)-2-hexen-1-ol. This is consistent with the increase in alcohol taste in the samples after multiple FT cycles. It is known that ethanol is relatively low in most fresh fruits, but it may accumulate in senescent fruits [37]. We speculate that the FT cycle destroys the blueberry tissue structure and promotes anaerobic respiration thereby accumulating ethanol in the juice [38]. In general, repeated FT cycles will increase the ethanol content of the blueberry and dilute or destroy the original green leafy flavor.

### 3.3. Effect of FT Cycles on Hot-Air Drying Kinetics of Blueberry Fruit

**Figure 5:**
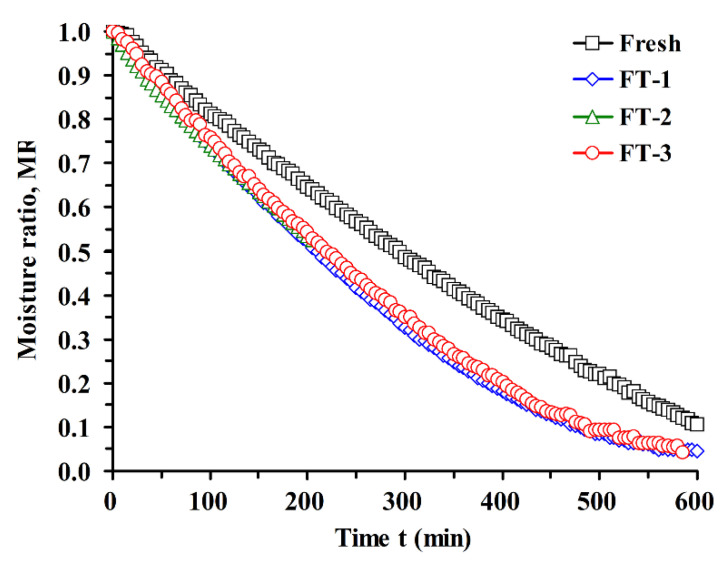
The hot-air drying curves of blueberry at 60 °C subjected to freeze-thaw (FT) treatments. FT-1, FT-2 and FT-3 represent the sample with FT treatment once, twice and third times.

Early results show that blueberry anthocyanins can remain stable below 60 °C [26,39]. Therefore, the hot-air temperature at 60 °C was selected to study the effect of freezing and thawing on the drying kinetics of blueberry. As predicted, freezing and thawing can shorten the hot-air drying time of blueberries. Figure 5 shows that the total time required to dry blueberry to 50% MR at 60 °C is about 290 min, while it only takes about 200 min for the FT-1 samples. So, one FT treatment can shorten the drying time by about 30% to achieve the same water content. Similar trends are found in the drying process of other agricultural products [7,40,41]. For example, the ultrasound freeze-thawing pretreatment reduced the drying time and also energy consumption in the drying of okra [40]. However, the samples after multiple FT cycles gave similar hot-air drying curves, which did not further improve the drying efficiency. From the perspective of energy consumption and costs, it is appropriate to choose one cycle of FT treatment for the drying of blueberry. The fitting results of nine models on the hot-air drying curve of blueberry at 60 °C are listed in Table S2 and Figure S3. The regression drying constants *a* and *b* of the Wang and Singh equation which gives the best results are shown in Table S3. The model and parameters will facilitate the calculation of hot-air drying of blueberry for further studies.

The *D_e_* value of the fresh blueberry sample is 2.69 × 10^−10^ m^2^/s, while the FT treated samples range between 4.43 × 10^−10^ and 4.59 × 10^−10^ m^2^/s, as shown in Table 3. The values are in the range of those reported for hot-air drying of different materials (10^−8^ to 10^−10^ m^2^/s) [41,42]. The *D_e_* values of the FT-treated sample are similar, which are about twice as large as the value of the fresh sample. This again means that the freeze-thawed promotes the diffusion rate of water in blueberries. It is known that freeze-thawing cycles can rupture cell walls and membrane, and change osmotic pressure, thereby promoted water transfer rate in the drying process [40]. Studies also found that the high *D_e_* values shown by FT treated samples were due to the formation of large ice crystals inside cells [40,41].

## 4. Conclusions

The effects of FT cycles on juice physicochemical properties, juice aroma profiles and hot-air drying kinetics of blueberry were studied. We found that the FT cycles will increase the juice yield of blueberries and reduce the anthocyanin content, while the pH and solids contents keep unchanged. (E)-2-hexenol, ethanol and 1-hexanol are characteristic substances of blueberry passing through FT cycles. The former gradually decreases, while the latter two gradually increase. The electronic nose shows the different aroma parents of FT-treated blueberries. The main volatile substances in the fresh juice are (E)-2-hexenal, α-terpineol, hexanal and linalyl formate, respectively. In the FT-treated samples, the amount of C6 aldehydes including (E)-2-hexenal and hexanal decreased significantly while the amount of α-terpineol and linalyl formate remained unchanged. Repeated FT cycles will increase the ethanol content of the blueberry. Finally, the drying kinetics of FT-treated blueberries was studied. FT treatment can shorten the drying time by about 30% to achieve the same water content. The *D_e_* values of the FT-treated sample are similar, which are about twice as large as the value of the fresh sample. The results will be beneficial for the processing of blueberry into juice or dried fruits.

## Figures and Tables

**Table 1:** Effect of freeze-thaw (FT) cycles on juice yield, pH and total soluble solids (TTS) of the juice. FT-1, FT-2 and FT-3 represent the sample with FT treatment once, twice and third times.

Sample	Juice Yield (g/g)	pH	TSS (°Brix)
Fresh	0.51 ± 0.00 ^d^	3.64 ± 0.02 ^c^	12.33 ± 0.33 ^a^
FT-1	0.57 ± 0.00 ^c^	3.70 ± 0.01 ^b^	12.33 ± 0.38 ^a^
FT-2	0.59 ± 0.00 ^b^	3.67 ± 0.03 ^bc^	12.73 ± 0.26 ^a^
FT-3	0.61 ± 0.00 ^a^	3.75 ± 0.03 ^a^	12.27 ± 0.19 ^a^

Values presented as mean ± standard deviation (*n* = 3). Different letters in each column denote significant differences (*p* < 0.05).

**Table 2:** Volatile organic compounds found in different freeze-thaw (FT) treated samples and its relative proportion (%) based on total ion chromatogram (TIC) peak areas in headspace gas chromatography-mass spectrometry. FT-1, FT-2 and FT-3 represent the sample with FT treatment once, twice and third times.

NAME	CAS	Relative Proportion (%)			
		Fresh	FT-1	FT-2	FT-3
ALCOHOLS					
Ethanol	64-17-5	0.81 ± 0.31 ^b^	5.48 ± 0.49 ^b^	38.61 ± 4.09 ^a^	43.46 ± 2.34 ^a^
(Z)-2-Hexen-1-ol	111-27-3	0.33 ± 0.06 ^c^	0.96 ± 0.09 ^c^	2.33 ± 0.12 ^b^	6.56 ± 0.24 ^a^
Eucalyptol	470-82-6	0.11 ± 0.08 ^a^	0.16 ± 0.02 ^a^	0.16 ± 0.09 ^a^	0.03 ± 0.04 ^a^
cis-Linalool oxide	5989-33-3	0.98 ± 0.28 ^b^	1.64 ± 0.29 ^a^	1.33 ± 0.33 ^a^^,b^	1.70 ± 0.07 ^a^
Hotrienol	20053-88-7	0.37 ± 0.02 ^a^	0.74 ± 0.29 ^a^	0.54 ± 0.11 ^a^	0.53 ± 0.15 ^a^
cis-Ocimenol	7643-59-6	0.47 ± 0.20 ^b^	0.73 ± 0.03 ^a^	0.69 ± 0.23 ^a^^,b^	0.87 ± 0.07 ^a^
Ocimenol	5986-38-9	0.26 ± 0.11 ^a^	0.38 ± 0.01 ^a^	0.40 ± 0.17 ^a^	0.38 ± 0.01 ^a^
trans-4-Thujanol	17699-16-0	0.33 ± 0.10 ^a^	0.51 ± 0.15 ^a^	0.37 ± 0.04 ^a^	0.26 ± 0.03 ^b^
α-Terpineol	98-55-5	17.56 ± 0.24 ^c^	36.70 ± 0.42 ^a^	23.90 ± 1.70 ^b^	20.67 ± 2.21 ^b^
p-Mentha-1(7),8-dien-2-ol	35907-10-9	0.11 ± 0.09 ^a^	0.23 ± 0.02 ^a^	0.14 ± 0.04 ^a^	0.20 ± 0.08 ^a^
ALDEHYDES					
Hexanal	66-25-1	13.99 ± 1.55 ^a^	1.88 ± 0.25 ^b^	2.37 ± 0.22 ^b^	2.22 ± 0.36 ^b^
(Z)-3-Hexenal	6789-80-6	0.86 ± 0.26 ^a^	0.15 ± 0.01 ^b^	0.08 ± 0.00 ^c^	0.07 ± 0.01 ^c^
(E)-2-Hexenal	6728-26-3	48.47 ± 0.10 ^a^	15.43 ± 2.13 ^b^	8.84 ± 0.38 ^c^	6.97 ± 0.58 ^c^
Carvomenthenal	29548-14-9	0.39 ± 0.08 ^a^	1.66 ± 1.55 ^a^	0.60 ± 0.24 ^a^	1.33 ± 0.76 ^a^
ESTERS					
Ethyl Acetate	141-78-6	0.37 ± 0.27 ^a^	0.44 ± 0.12 ^a^	0.23 ± 0.33 ^a^	0.54 ± 0.50 ^a^
Linalyl formate	115-99-1	7.81 ± 2.70 ^b^	18.96 ± 4.11 ^a^	12.00 ± 3.04 ^a^	9.43 ± 0.83 ^b^
3-Cyclohexen-1-ol, 5-methylene-6-(1-methylethenyl)-, acetate	54832-23-4	1.32 ± 0.32 ^ab^	2.68 ± 1.12 ^a^	1.25 ± 0.10 ^b^	1.48 ± 0.06 ^a^
HYDROCARBONS					
o-Cymene	527-84-4	0.35 ± 0.06 ^b^	0.52 ± 0.02 ^a^	0.22 ± 0.04 ^c^	0.10 ± 0.01 ^d^
p-Cymenene	1195-32-0	0.83 ± 0.17 ^b^	1.47 ± 0.10 ^a^	0.96 ± 0.12 ^b^	0.60 ± 0.00 ^c^
TERPENES					
γ-Ionone	79-76-5	0.14 ± 0.01 ^b^	0.39 ± 0.03 ^a^	0.11 ± 0.05 ^bc^	0.07 ± 0.00 ^c^
β-Myrcene	123-35-3	0.07 ± 0.03 ^b^	0.22 ± 0.03 ^a^	0.10 ± 0.01 ^b^	0.11 ± 0.09 ^a^^b^
α-Terpinene	99-86-5	0.57 ± 0.13 ^b^^c^	1.19 ± 0.07 ^a^	0.71 ± 0.03 ^b^	0.41 ± 0.0 ^c^
D-Limonene	5989-27-5	0.76 ± 0.03 ^b^	1.64 ± 0.29 ^a^	0.91 ± 0.13 ^b^	0.35 ± 0.00 ^c^
1S-α-Pinene	7785-26-4	0.22 ± 0.01 ^b^	0.49 ± 0.19 ^a^	0.25 ± 0.03 ^b^	0.05 ± 0.01 ^c^
3-Carene	13466-78-9	0.39 ± 0.00 ^b^	0.87 ± 0.29 ^a^	0.36 ± 0.21 ^bc^	0.18 ± 0.01 ^c^
γ-Terpinene	99-85-4	0.15 ± 0.04 ^b^	0.32 ± 0.09 ^a^	0.14 ± 0.03 ^b^	0.03 ± 0.05 ^c^
Terpinolene	586-62-9	1.30 ± 0.10 ^b^	3.23 ± 0.42 ^a^	1.75 ± 0.24 ^b^	0.79 ± 0.00 ^c^
L-β-Pinene	18172-67-3	0.68 ± 0.07 ^a^	0.93 ± 0.33 ^a^	0.64 ± 0.07 ^a^	0.62 ± 0.17 ^a^

Values presented as mean ± standard deviation (*n* = 2). Different letters in each row denote significant differences (*p* < 0.05).

**Table 3:** The effective moisture diffusivity (*D_e_*, m^2^/s) of raw blueberry and freeze-thaw (FT) treated blueberry fruit at 60 °C. FT-1, FT-2 and FT-3 represent the sample with FT treatment once, twice and third times.

Samples	Effective Diffusivity, *D_e_* (m^2^/s)	R^2^
Fresh	2.69 × 10^−10^	0.962
FT-1	4.43 × 10^−10^	0.973
FT-2	4.68 × 10^−10^	0.955
FT-3	4.59 × 10^−10^	0.964

## Data Availability

Data sharing is not applicable to this article.

## Supplementary Materials

The following are available online at https://www.mdpi.com/article/10.3390/foods10102362/s1, Figure S1: The top-down pictures represent the GC-MS base peak chromatogram of the total ion chromatogram (TIC) for the fresh, FT-1, FT-2 and FT-3 samples. FT-1, FT-2 and FT-3 represent the blueberry with freeze-thaw (FT) treatment once, twice and third times; Figure S2: Peak area in TIC assigned to each compound for (A) fresh juice, (B) freeze-thaw (FT) once, (C) FT twice and (D) FT third times; Figure S3: The correlation coefficient of a different model for the fitting of the hot-air drying curve of frozen blueberry. FT-1, FT-2 and FT-3 represent the blueberry with freeze-thaw (FT) treatment once, twice and third times; Table S1: Mathematical models applied to fit the drying curves; Table S2: The fitting models and statistical results for models. FT-1, FT-2 and FT-3 represent the blueberry with freeze-thaw (FT) treatment once, twice and third times; Table S3: Parameter of the Wang and Singh model for the correlation of hot-air drying curve of freeze-thaw (FT) treated blueberry fruit at 60 °C. FT-1, FT-2 and FT-3 represent the sample with FT treatment once, twice and third times.
