# Supplementary material for: Effect of Freeze-Thaw Cycles on Juice Properties, Volatile Compounds and Hot-Air Drying Kinetics of Blueberry
Source: Foods. 2021 Oct 4;10(10):2362. doi: 10.3390/foods10102362 (PMC8535103; doi:10.3390/foods10102362)
Supplement: Supplementary file 1 [file foods-10-02362-s001.zip › foods-1324018-supplementary.pdf]

Supplementary Materials

Table S1. Mathematical models applied to fit the drying curves.

**Equation**	**Model name**	**Parameters**
$MR = \exp(-k\times t)$	Newton	k
$MR = \exp(-k\times t^n)$	Page	k, n
$MR = \exp[-(k\times t)^n]$	Modified page	k, n
$MR = a\times\exp(-k\times t)$	Henderson and Pabis	a, k
$MR = 1+a\times t+b\times t^2$	Wang and Singh	a, b
$MR = a\times\exp(-k\times t) + (1-a)\times\exp(-k\times a\times t)$	Two term exponential	a, k
$MR = a\times\exp(-k\times t) + c$	Logarithmic	a, k, c
$MR = a\times\exp(-k_0\times t) + \exp(-k_1\times t)$	Two-term	a, k_0, k_1
$MR = a\times\exp(-k\times t) + (1-a)\times\exp(-k\times b\times t)$	Diffusion approach	a, b, k

Table S2. The fitting models and statistical results for models. FT-1, FT-2, and FT-3 represent the blueberry with freeze-thaw (FT) treatment once, twice, and third times.

**Model Name**	**Fresh**		**FT-1**		**FT-2**		**FT-3**	
	R^2	**RMSE**	R^2	**RMSE**	R^2	**RMSE**	R^2	**RMSE**
Newton	0.966	0.002361	0.973	0.002264	0.969	0.002636	0.969	0.002703
Page	0.995	0.000329	0.997	0.000262	0.993	0.000562	0.996	0.000304
Modified page	0.995	0.000329	0.997	0.000262	0.993	0.000562	0.996	0.000304
Henderson and Pabis	0.979	0.001524	0.983	0.001493	0.976	0.002031	0.980	0.001745
Wang and Singh	1.000	0.000016	1.000	0.000041	0.999	0.000052	0.999	0.000080
Two term exponential	0.994	0.000411	0.996	0.000359	0.992	0.000695	0.995	0.000375
Logarithmic	1.000	0.000010	0.998	0.000153	0.998	0.000133	0.998	0.000142
Two-term	0.848	0.010827	0.876	0.010767	0.870	0.011155	0.872	0.011179
Diffusion approach	0.995	0.000351	0.997	0.000284	0.993	0.000592	0.996	0.000358

Table S3. Parameter of the Wang and Singh model for the correlation of hot-air drying curve of freeze-thaw (FT) treated blueberry fruit at 60 °C. FT-1, FT-2, and FT-3 represent the sample with FT treatment once, twice, and third times.

**Sample**	**a (min^{-1})**	**b (min^{-2})**	R^2	**RMSE**
Fresh	-1.902×10^{-3}	6.78×10^{-7}	1.000	1.6×10^{-5}
FT-1	-2.856×10^{-3}	2.00×10^{-6}	1.000	4.1×10^{-5}
FT-2	-2.733×10^{-3}	1.83×10^{-6}	0.999	5.2×10^{-5}
FT-3	-2.691×10^{-3}	1.78×10^{-6}	0.999	8.0×10^{-5}

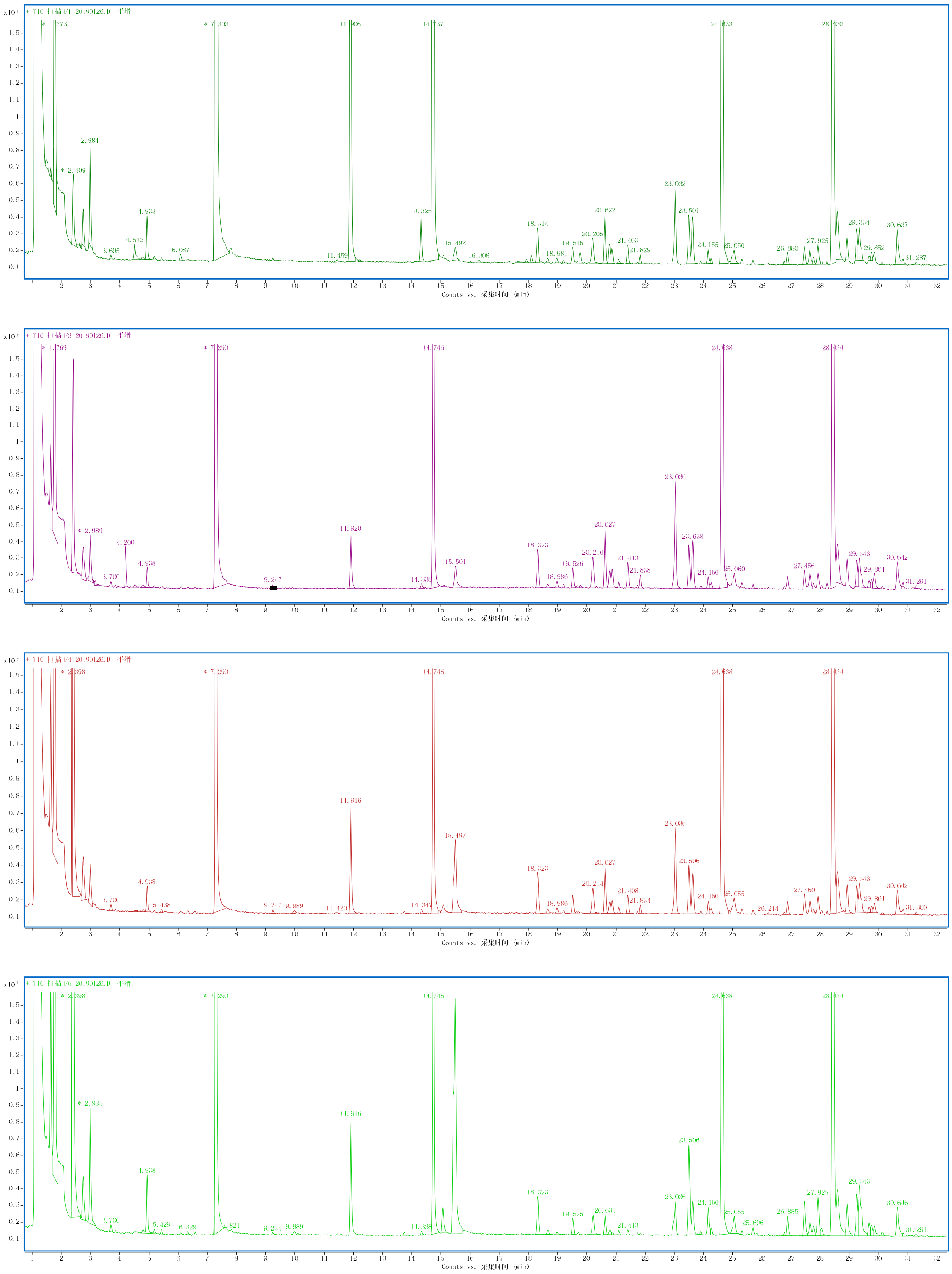

Figure S1. The top-down pictures represent the GC-MS base peak chromatogram of the total ion chromatogram (TIC) for the fresh, FT-1, FT-2 and FT-3 samples. FT-1, FT-2, and FT-3 represent the blueberry with freeze-thaw (FT) treatment once, twice, and third times.

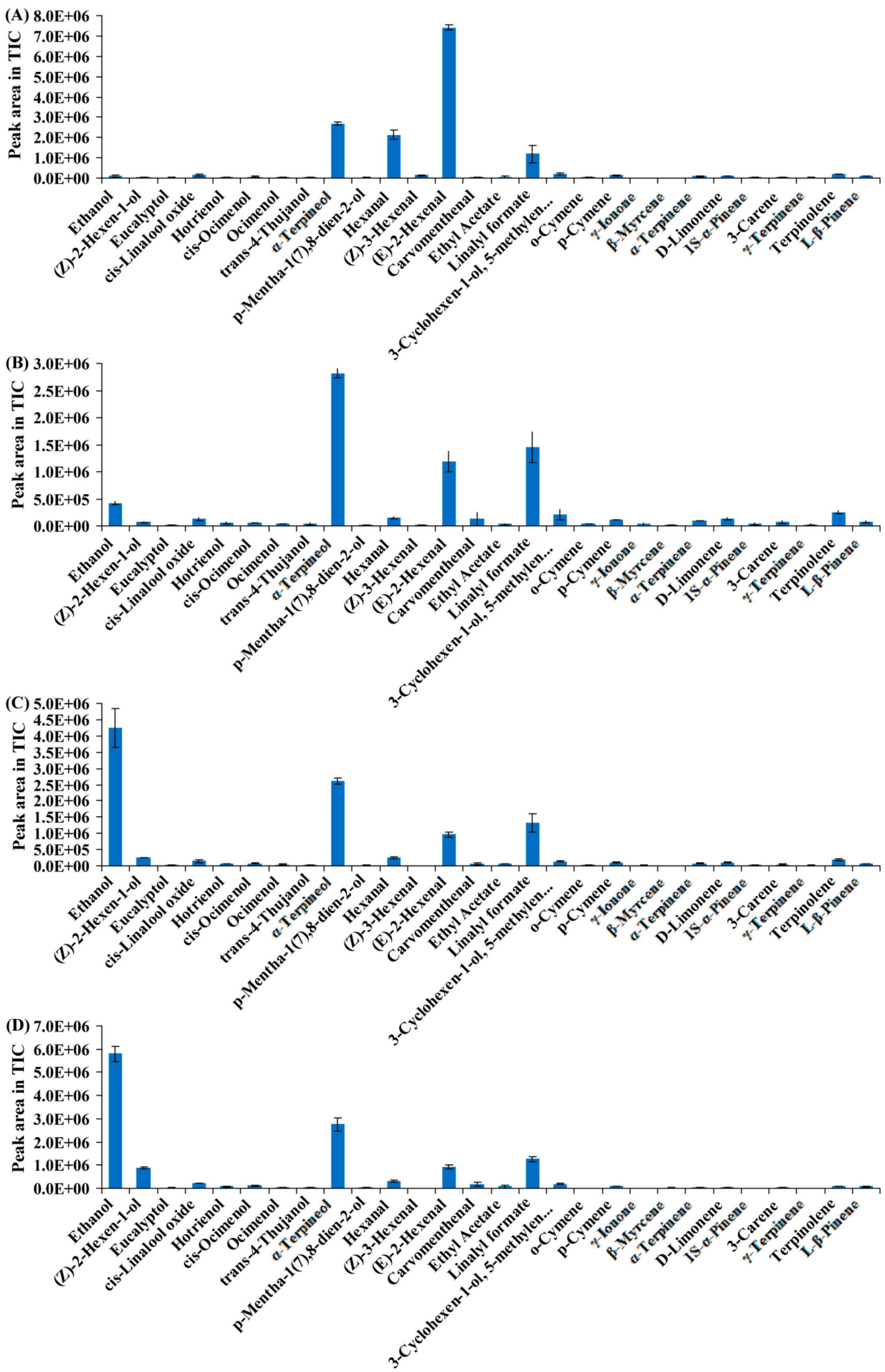

Figure S2. Peak area in TIC assigned to each compound for (A) fresh juice, (B) freeze-thaw (FT) once, (C) FT twice, and (D) FT third times.

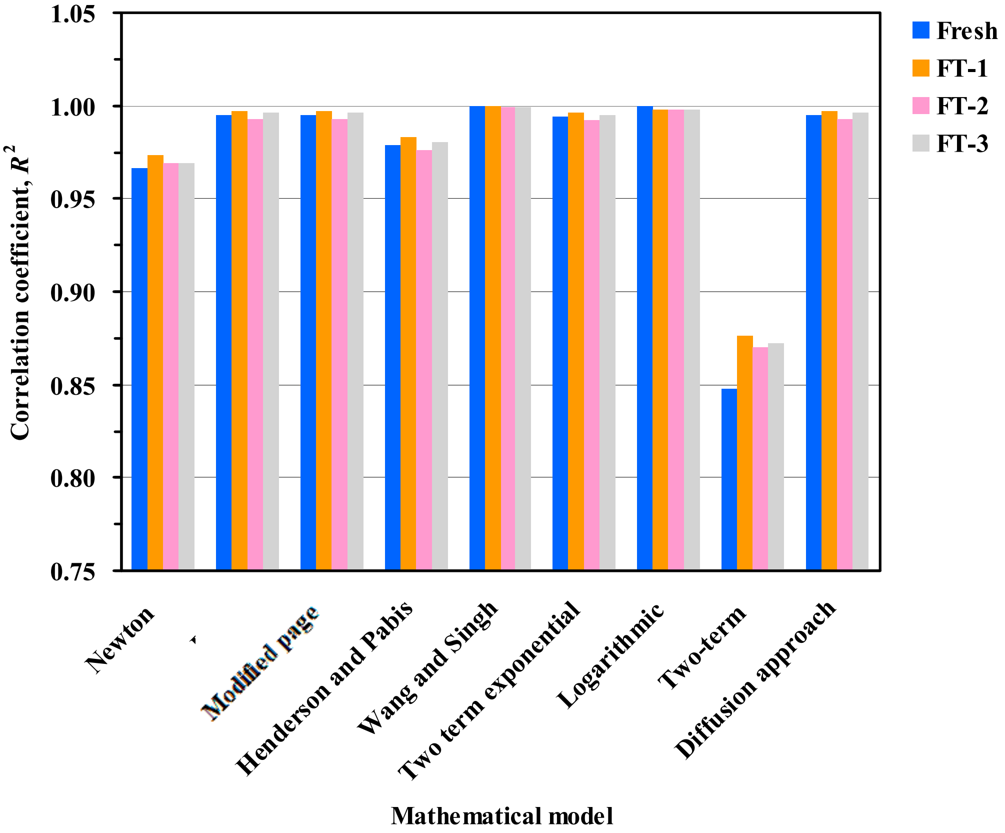

Figure S3. The correlation coefficient of a different model for the fitting of the hot-air drying curve of frozen blueberry. FT-1, FT-2, and FT-3 represent the blueberry with freeze-thaw (FT) treatment once, twice, and third times.
